# Autologous omentum transposition for regeneration of a renal injury model in rats

**DOI:** 10.1186/s40779-021-00361-0

**Published:** 2022-01-04

**Authors:** Tayfun Bilgiç, Ümit İnce, Fehmi Narter

**Affiliations:** 1 Acıbadem Kadıkoy Hospital of General Surgery, Istanbul, 34718 Turkey; 2 Department of Pathology, Acıbadem Mehmet Ali Aydınlar University, Istanbul, 34684 Turkey; 3 Department of Urology, Acıbadem Mehmet Ali Aydınlar University, Istanbul, 34684 Turkey

**Keywords:** Omentum, Rat, Regeneration, Renal trauma

## Abstract

**Background:**

After renal trauma, surgical treatment is vital, but sometimes there may be loss of function due to fibrosis. This study aimed to evaluate the effect of autologous omentum flaps on injured renal tissues in a rat model.

**Methods:**

A total of 30 Wistar albino rats were included and randomly divided equally into a control group and four intervention groups. Iatrogenic renal injuries were repaired using a surgical technique (primary repair 1 group and primary repair 2 group) or transposition of the autologous omentum (omentum repair 1 group and omentum repair 2 group). Blood samples were taken preoperatively and on the 1st and 7th postoperative days in all groups and on the 18th postoperative day in the control and two intervention groups. All rats were sacrificed on the 7th or 18th day postoperatively, and their right kidneys were taken for histopathological evaluation.

**Results:**

The mean urea level significantly decreased from day 1 to day 7 and from day 1 to day 18 in the omentum repair 2 group (*P* = 0.005 and *P* = 0.004, respectively). There were no other significant changes in urea or creatinine levels within the intervention groups (*P* > 0.05). There was no significant correlation between the urea and creatinine levels and the histological scores (*P* > 0.05). The primary repair 1 and 2 groups had significantly higher median granulation and inflammation scores in the kidney specimen than the control and omentum repair groups (*P* < 0.05). The omentum repair 2 group had significantly lower median granulation and inflammation scores in the surrounding tissues than the primary repair 2 group (*P* < 0.05). The completion score for the healing process in the kidney specimen was significantly higher in the omentum repair groups than in the primary repair groups (*P* < 0.05). The omentum repair 2 group had significantly lower median granulation and inflammation scores in the surrounding tissues than the primary repair 2 group (*P* < 0.05). Granulation degree in the kidney specimen was strongly and positively correlated with the inflammation degree (*r* = 0.824, *P* < 0.001) and foreign body reaction in the kidney specimen (*r* = 0.872, *P* < 0.001) and a strong and negative correlation with the healing process completion score in the kidney (*r* = − 0.627, *P* = 0.001). Inflammation degree in the kidney specimen was strongly and positively correlated with the foreign body reaction in the kidney specimen (*r* = 0.731, *P* = 0.001) and strongly and negatively correlated with the healing process completion score in the kidney specimen (*r* = − 0.608, *P* = 0.002).

**Conclusion:**

Autologous omentum tissue for kidney injury repair attenuated inflammation and granulation. Additionally, the use of omental tissue to facilitate healing of kidney injury may theoretically lead to a more effective healing process and reduced fibrosis and tissue and function loss.

## Background

Renal trauma can occur through various mechanisms. Etiological factors are generally described as blunt kidney injuries (80–90%) and penetrating kidney injuries (10%). Although not always apparent, hematuria is the main symptom of kidney trauma [1]. The main purpose of treatment is to ensure that the kidney function returns to normal as soon as possible [2]. Renal injuries are classified into 5 grades according to severity. In particular, grade 4–5 injuries in renal tissues are a candidate for surgical treatment. Major kidney injuries caused by blunt trauma are treated with conservative management [2, 3]. The other treatment approaches are open or endoscopic surgical procedures such as laparoscopic/robot-assisted or open partial/total nephrectomy, nephrorraphy, autotransplantation, and embolization.

It is important to accelerate wound healing in renal traumas requiring surgical treatment to decrease the morbidity and mortality rates. Wound healing includes three dynamic phases: inflammation, proliferation, and remodeling [4]. Angiogenesis, inflammation, cellular proliferation, collagenization, granulation, and epithelialization are important processes in the remodeling of tissues [5]. Many molecules, such as vascular endothelial growth factor (VEGF) and nitric oxide (NO), play a role in tissue regeneration [6]. Synthetic or autologous materials such as fat tissue, omentum, meshes, and fascias can be used for the regeneration of injured kidney tissue [7].

The omentum is a vascular, fatty structure and its progenitor cells produce many growth and angiogenic factors [8, 9]. Therefore, it can migrate to damaged tissues and aid in the regeneration process [10, 11]. It has been used for many surgical procedures, such as the treatment of bone fractures, spine injuries, ischemic heart diseases, and hepatic injuries [12]. Progenitor stem cells have high proliferation and differentiation capabilities. However, they have a very short lifespan in the tissue after they are injected. In many studies, the omentum has been used to wrap the injured tissue, and it has been shown to be useful in regeneration [13]. According to the results of a previous study, progression to chronic kidney failure has been shown to slow down after partial nephrectomy when the omentum was used to cover the kidneys [11]. This is a unique contribution in the context of nephron sparing. This study aimed to evaluate the repair effect of the transposition of the autologous omentum on injured renal tissues in a rat model.

## Methods

### Animals and groups

A total of 30 Wistar albino rats of the same sex and similar age and the same weight of 250–300 g were used. The investigation only used one sex in order to maintain a consistent and standardized dataset. The animals were randomly divided into a control group and four intervention groups: primary repair 1 group, primary repair 2 group, omentum repair 1 group, and omentum repair 2 group, with 6 rats in each. The animal experiments were approved by the Turkish Medicines and Medical Devices Agency and the Local Ethical Committee on Animal Experiments (Acıbadem Mehmet Ali Aydınlar University, ACU-HADYEK 2018/47).

### Animal experiments

In all intervention groups, 8-mm diameter and 4-mm deep parenchymal damage was generated with Stiefel biopsy forceps on the front surface of the right kidneys according to the well-described Stiefel biopsy technique [14]. In the primary repair groups, kidney injuries were primarily repaired with the interrupted atraumatic matrix suture technique (Ethicon VICRYL Rapid 8–0, fastest absorbable, synthetic, braided, composed of a copolymer made from 90% glycolide and 10% L-lactide, absorption time 7–10 days). In the omentum repair groups, transposition of the autologous omentum was used without primary sutures on the injured renal tissue for repair. A sham operation was performed on the rats in the control group. We selected the time of sacrifice as the 18th postoperative day based on a previous study [14], which reported that posttraumatic necrosis in the tissue disappeared on the 18th day. In that study, after this period, collagen maturation took place in the renal capsule, and the connective tissue at the edges of the wound was contracted.

All rats were kept in standardized laboratory conditions of 20–24 °C, 50–60% relative humidity, controlled light (day/night cycle of 12 h/12 h), fed standardized rodent food, and given filtered and chlorinated water. The animals were anesthetized with an intraperitoneal injection of ketamine (75 mg/kg, Pfizer) and xylazine (5 mg/kg, Bioveta). All rats were protected against postoperative infections with an antibiotic (cefazolin 15 mg/kg, SC). The only exclusion criterion for this study was the death of the rats before the end of the study.

In all groups, blood samples were taken preoperatively on the 1st and 7th postoperative days for creatinine and urea analyses. Additional blood samples were obtained on the 18th postoperative day for the same analyses in the control, primary repair 2, and omentum repair 2 groups.

All rats in the control, primary repair 2, and omentum repair 2 groups were sacrificed on the 18th postoperative day, and their right kidneys were taken for histopathological evaluation. The rats in the primary repair 1 and omentum repair 1 groups were sacrificed on the 7th postoperative day, and their right kidneys were taken for histopathological evaluation.

### Blood biochemical analysis

The concentrations of creatinine and urea in the serum were determined by an enzymatic assay (Roche Diagnostics GmbH, Mannheim, Germany). Serum samples for the measurement were collected and stored at − 80 °C until the analysis was carried out. All laboratory investigators were blinded to each rat’s clinical information.

### Tissue sampling and histopathological examination

All kidney samples were fixed in a 10% formaldehyde solution. Kidney tissues were embedded in paraffin, and 5 μm tissue sections were obtained for hematoxylin–eosin (H&E) and Masson’s trichrome (MTC) staining protocols for collagen fibers. In addition to the macroscopic view, the histopathological evaluation consisted of granulation, inflammation, fibrosis, foreign body reaction, and healing in the injured kidney and surrounding tissue (omentum). All components were scored between 0 and 5 according to the density of the changes in the tissue (normal: 0, rare: 1, mild: 2, modest: 3, common: 4, and excessive: 5). The macroscopic evaluation consisted only of a macroscopic view of the kidney to review the surface of the kidney in terms of the presence of abnormal structures, and it was performed with a quantitative/semi-quantitative analysis. The degree of granulation was evaluated in the glomeruli and parenchymal tissue and by reviewing these structures in terms of edema, inflammatory cells, angiogenesis, and fibroblasts. Inflammation was evaluated by reviewing the tissues regarding acute inflammatory cells, macrophages, and lymphocytes. The degree of fibrosis (connective tissue evaluation) was evaluated by the presence of fibroblasts and their density. Foreign body reaction was evaluated with the following: necrosis, erythrocytes, and chronic inflammation findings. Healing was determined by the findings of regeneration and normalization in the tissues. The cut sections were examined for completeness, and one representative section of each kidney was selected for tissue processing. The histological damage was examined under a light microscope by a pathologist who was blinded to the study design (sham vs. renal regeneration). All pathological slides were scanned using a digital pathology system (3D Histech Company, P250—Flash III Digital Scanner, 20X), and microscopic photos were taken using software (3D Histech company, CaseViewer software).

The combined morphologic score was calculated for the renal tissue and surrounding tissues as follows: Microscopy score 5, granulation score 5, inflammation score 5, connective tissue score 5, foreign body score 5, and healing score 5.

### Statistical analysis

SPSS 21.0 (IBM Corp., Armonk, NY, USA) software was used for statistical analysis. It was estimated that 5 groups comprising 6 rats per group would be required to detect 3 units of improvement [with 1.5 units as the standard deviation (SD)] as a significant effect in a wound healing model, assuming a power of 80% and a confidence level of 95%. The descriptive statistics for categorical variables are given as *n* (%). Continuous variables with non-normal distribution are presented as median [interquartile range (IQR)], and continuous variables with normal distribution are presented as the means ± SD. The one sample Kolmogorov–Smirnov test was used to assess the normality of the variables. Variables with skewed distribution were compared using the Kruskal–Wallis and Mann–Whitney *U* tests. Variables with normal distribution were analyzed using ANOVA. Post-hoc analysis was performed using Tukey's range test. Dependent variables with normal distribution were analyzed using Pearson tests. Spearman’s test was used for correlation analyses. Statistical significance was defined as *P* < 0.05.

## Results

### Comparison of urea and creatinine levels

The mean creatinine level decreased from day 1 to day 7 in the control group, but the difference was not significant (*P* = 0.987). The mean creatinine and urea levels decreased from day 1 to day 7 in the primary repair 1 group, but the differences were not significant (*P* = 0.401 and *P* = 0.070, respectively). In the primary repair 2 group, the mean creatinine level decreased significantly from day 1 to day 7 and day 1 to day 18 but the differences were not significant (*P* = 0.401 and *P* = 0.776, respectively). There was no significant change in mean urea or creatinine levels in the omentum repair 1 group (*P* > 0.05). The mean urea level significantly decreased from day 1 to day 7 and from day 1 to day 18 in the omentum repair 2 group (*P* = 0.005 and *P* = 0.004, respectively). There were no other significant changes in urea or creatinine levels within the intervention groups (*P* > 0.05, Table 1).

**Table 1:** Comparison of creatinine and urea levels in each group [mg/dl, mean ± SD]

Item	Day 1	Day 7	Day 18	*P* value*	*P* value^#^
Creatinine					
Control group	0.270 ± 0.041	0.230 ± 0.063	0.270 ± 0.033	0.987	1.000
Primary repair 1 group	0.360 ± 0.043	0.270 ± 0.022	NA	0.401	
Primary repair 2 group	0.290 ± 0.039	0.200 ± 0.027	0.220 ± 0.020	0.401	0.776
Omentum repair 1 group	0.280 ± 0.037	0.360 ± 0.141	NA	0.656	
Omentum repair 2 group	0.270 ± 0.037	0.230 ± 0.086	0.270 ± 0.088	0.997	1.000
Urea					
Control group	43.670 ± 5.279	38.000 ± 3.742	36.170 ± 2.401	0.494	0.117
Primary repair 1 group	39.330 ± 3.933	31.330 ± 3.077	NA	0.070	
Primary repair 2 group	43.330 ± 5.574	37.170 ± 3.312	39.670 ± 4.761	0.360	0.947
Omentum repair 1 group	35.500 ± 2.881	33.170 ± 4.355	NA	0.999	
Omentum repair 2 group	43.170 ± 5.270	33.000 ± 5.367	32.830 ± 2.563	0.005	0.004

*NA* not available

*Day 7 vs. day 1

^#^Day 18 vs. day 1

### Comparison of histopathological variables

**Fig. 1:**
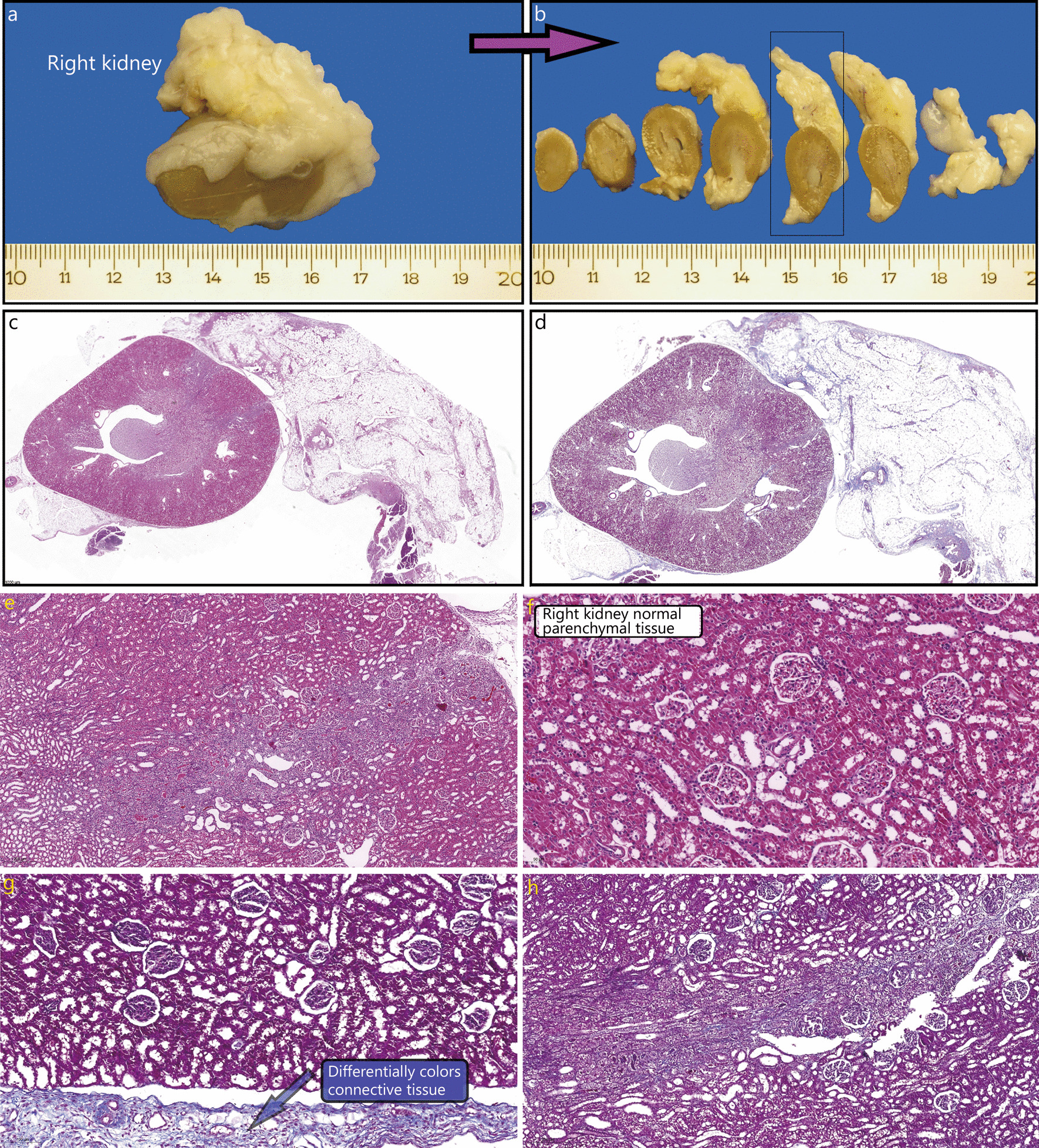
Sample macroscopic and microscopic images. **a**, **b** Macroscopic view of the kidney and omentum. Arrow indicates that the kidney piece shown on the left side was sliced as in the figure on the right. **c** Cross-section of the whole kidney (4 µm) (HE × 57.6). **d** Right bottom, Cross-section of the whole kidney (4 µm) (Masson trichrome staining × 30). **e** Kidney, parenchyma, trauma line—microscopically (HE × 380). **f** Kidney, parenchyma—microscopically (HE × 1120). **g** Kidney, parenchyma, trauma line—microscopically (Masson trichrome staining × 256). **h** Kidney, parenchyma, surrounding area—microscopically (Masson trichrome staining × 440)

Examples of histopathological changes are shown in Fig. 1, and combined scores of morphologic evaluation in the renal and surrounding tissue are shown in Table 2. The primary repair groups had higher combined histological scores for renal and surrounding tissues than the control group (*P* < 0.05). The omental repair groups had similar combined histological scores for renal tissue (*P* > 0.05) and higher combined histological scores for surrounding tissues (*P* < 0.05) than the control group (Table 2).

**Table 2:** Comparison of the combined histological scores in each group (*n* = 6)

Group	Combined morphologic score for renal tissue		Combined morphologic score for surrounding tissue	
	Mean ± SD	95% CI	Mean ± SD	95% CI
Control group*	2.233 ± 0.151	2.075–2.391	1.100 ± 0.276	0.811–1.389
Primary repair 1 group^#^	3.567 ± 0.234^a^	3.321–3.812	2.367 ± 0.151^a^	2.209–2.525
Primary repair 2 group*	3.000 ± 0.537^a,b^	2.437–3.563	2.333 ± 0.207^a^	2.117–2.550
Omentum repair 1 group^#^	2.300 ± 0.245^b,c^	2.043–2.557	2.000 ± 0.506^a^	1.469–2.531
Omentum repair 2 group*	2.367 ± 0.266^b,c^	2.088–2.646	1.667 ± 0.163^a,b,c^	1.495–1.838

*SD* standard deviation, *CI* confidence interval

*Postoperative day 18 sacrifice

^#^Postoperative day 7 sacrifice

^a^*P* < 0.05, compared with the control group

^b^*P* < 0.05, compared with the primary repair 1 group

^c^*P* < 0.05, compared with the primary repair 2 group

The primary repair 1 and 2 groups had significantly higher median granulation and inflammation scores in the kidney specimen than the control and omentum repair groups (*P* < 0.05). The omentum repair groups had granulation and inflammation scores similar to those of the control group (*P* > 0.05). The foreign body reaction score in the kidney specimen was significantly higher in the primary repair groups than in the control group (*P* < 0.05). The completion score for the healing process in the kidney specimen was significantly higher in the omentum repair groups than in the primary repair groups (*P* < 0.05).

The primary repair groups and omentum repair groups had a significantly higher median foreign body reaction score and connective tissue fibrosis score in the surrounding tissues than the control group (*P* < 0.05). The omentum repair 2 group had significantly lower median granulation and inflammation scores in the surrounding tissues than the primary repair 2 group (*P* = 0.005 and *P* = 0.006, respectively). The details of the histopathological score comparisons are given in Table 3.

**Table 3:** Comparison of the histopathological variables in each group [median (IQR)]

Item	Control group*	Primary repair 1 group^#^	Primary repair 2 group*	Omentum repair 1 group^#^	Omentum repair 2 group*
Kidney (right)					
Macroscopy	5 (5, 5)	5 (4, 5)	5 (3.75, 5)	5 (4, 5)	5 (4.75, 5)
Granulation	0 (0, 0)	3 (3, 4)^a^	1 (0.75, 2)^a^	0 (0, 0)^b^	0 (0, 0)^c^
Inflammation	0 (0, 1)	3 (3, 3.25)^a^	2.5 (2, 4)^a^	0.5 (0, 1)^b^	0.5 (0, 1.25)^c^
Connective tissue fibrosis	1 (0.75, 1)	1 (1, 1)	1 (1, 2)	1 (1, 2)	1 (1, 2)
Foreign body reaction	0 (0, 0)	2 (1, 2)^a^	1 (1, 1.25)^a^	–	–
Healing	–	4 (3.75, 4.25)	4 (3, 5)	5 (5, 5)^b^	5 (5, 5)
Surrounding tissue					
Granulation	2 (1.75, 2.25)	2.5 (2, 3)	2.5 (2, 3)	1.5 (1, 2.25)	1 (1, 1.25)^c^
Inflammation	2 (2, 3.25)	3 (3, 4)	3 (2.75, 3.25)	3 (2, 3.25)	2 (2, 2)^c^
Connective tissue fibrosis	1 (1, 1)	2 (2, 2)^a^	2 (1.75, 2)^a^	2 (1, 3)^a^	2 (1.75, 2)^a^
Foreign body reaction	0 (0, 0)	4 (4, 4)^a^	4 (4, 5)^a^	3.5 (3, 4)^a^	3 (3, 4)^a,c^

– No data

*Postoperative day 18 sacrifice

^#^Postoperative day 7 sacrifice

^a^*P* < 0.05, compared with the control group

^b^*P* < 0.05, compared with the primary repair 1 group

^c^*P* < 0.05, compared with the primary repair 2 group

### Correlation analysis

There were moderate to strong positive correlations between granulation and inflammation (*r* = 0.490, *P* = 0.006), inflammation and fibrosis (*r* = 0.397, *P* = 0.030), inflammation and foreign body reaction (*r* = 0.431, *P* = 0.017), and foreign body reaction and fibrosis in the surrounding tissue (*r* = 0.708, *P* < 0.001) (Table 4). Macroscopy of the kidney specimen, urea level, or creatinine level did not have any significant correlations with any of the study variables (*P* > 0.05). In the kidney specimen, there were strong correlations between granulation and inflammation (*r* = 0.824, *P* < 0.001), granulation and foreign body reaction (*r* = 0.872, *P* < 0.001), and inflammation and foreign body reaction (*r* = 0.731, *P* = 0.001). Healing process completion was inversely correlated with granulation (*r* = − 0.627, *P* = 0.001) and inflammation (*r* = − 0.608, *P* = 0.002) in the kidney specimen. However, the fibrosis degree in the kidney specimen was correlated only with the fibrosis degree in the surrounding tissue (*r* = 0.429, *P* = 0.018).

**Table 4:** Correlation analyses between study variables

Variables		Surrounding tissue				Kidney specimen					
		Granulation	Inflammation	Fibrosis	Foreign body reaction	Macroscopy	Granulation	Inflammation	Fibrosis	Foreign body reaction	Healing process completion
Inflammation degree in surrounding tissue	*r*	0.490									
	*P*	0.006									
Fibrosis degree in surrounding tissue	*r*	0.202	0.397								
	*P*	0.284	0.030								
Foreign body reaction in surrounding tissue	*r*	0.336	0.431	0.708							
	*P*	0.070	0.017	< 0.001							
Macroscopy of the kidney specimen	*r*	− 0.048	0.110	0.166	− 0.169						
	*P*	0.800	0.564	0.380	0.372						
Granulation degree in kidney specimen	*r*	0.478	0.591	0.394	0.635	− 0.073					
	*P*	0.008	0.001	0.031	< 0.001	0.702					
Inflammation degree in kidney specimen	*r*	0.512	0.507	0.434	0.660	− 0.018	0.824				
	*P*	0.004	0.004	0.017	< 0.001	0.926	< 0.001				
Fibrosis degree in kidney specimen	*r*	0.199	0.057	0.429	0.094	0.043	− 0.066	0.120			
	*P*	0.293	0.763	0.018	0.621	0.822	0.731	0.528			
Foreign body reaction in kidney specimen	*r*	0.273	0.323	0.829	0.813	− 0.363	0.872	0.731	0.191		
	*P*	0.272	0.192	< 0.001	< 0.001	0.139	< 0.001	0.001	0.448		
Healing process completion in kidney	*r*	− 0.625	− 0.305	0.076	− 0.425	0.267	− 0.627	− 0.608	0.024	0.000	
	*P*	0.001	0.148	0.724	0.039	0.208	0.001	0.002	0.910	1.000	
Urea level in blood	*r*	0.012	− 0.076	− 0.265	− 0.156	0.092	− 0.143	− 0.062	0.007	-0.205	− 0.207
	*P*	0.952	0.691	0.157	0.411	0.628	0.450	0.743	0.970	0.415	0.331
Creatinine level in blood	*r*	− 0.296	− 0.018	− 0.024	− 0.340	− 0.023	− 0.233	− 0.321	-0.052	-0.064	0.338
	*P*	0.112	0.925	0.899	0.066	0.905	0.215	0.084	0.784	0.801	0.106

## Discussion

The prevalence of renal trauma ranges between 0.3 and 3.25% in the literature, and the most common causes are blunt trauma followed by penetrating trauma. The most common renal trauma classification is the American Association for the Surgery of Trauma (AAST) classification, with grades 1–5 [15]. Currently, except for hemodynamically unstable grade 4–5 renal trauma, renal injuries are followed up with a conservative approach. Surgical intervention is also considered in cases of significant vital changes related to renal injury.

Partial/total nephrectomy or nephrorraphy can be selected according to the type or degree of injury. Usually, the transperitoneal surgical approach is preferable because this route provides some advantages, such as the early control of large veins and arteries. Surgery for renal trauma comprises control of the bleeding by sutures, watertight closure of the collecting system, and closure of parenchymal injuries. Even preserving thirty percent of kidney capacity can provide adequate kidney function, and the renal capsule should be preserved in all possible cases for successful repair [16]. Sometimes, if the renal capsule is not available, a pedicle flap of the omentum, free peritoneal graft, free fat graft, or polyglycolic acid mesh can be used for coverage of a large defect. In this technique, the omentum is placed on the injured tissue and superficially sutured with monofilament absorbable sutures [17–19].

The omentum has long been known to have the capacity to migrate to injured organs such as bones, spinal cords, heart, liver, and pancreas and facilitate healing. Many studies have shown that a reduction in total nephron capacity may cause kidney failure in the future; thus, maximum protection of kidney tissue should be the main goal. Some suture materials and surgical techniques can be harmful to kidney tissue. For this reason, alternative techniques have been developed to better protect the kidney tissue, especially in cases of large amounts of tissue loss. One of them is to use the omentum or fatty tissue to repair the renal injury.

Mesenchymal stem cells (MSCs) can be obtained from adipose tissue, peripheral blood, or bone marrow. Another alternative source for repairing injured tissue is the omentum. It is a very vascular structure and is suitable to facilitate repair in case of injury, as it contains a large number of growth and angiogenic factors and progenitor cells for regeneration [20]. MSCs were first isolated from adipose tissue in 2001 by Zuk et al. [19]. It is well known that MSCs have multipotency, self-renewal, proliferation, regeneration, and differentiation abilities [20]. Of note, MSCs can accelerate tissue repair by direct migration to injured sites [21, 22]. Alternatively, MSCs may be administered locally or systemically for treatment. It is widely agreed that transplanted MSCs can directly reconstruct impaired organs. They have some specific features, such as endocrine (growth factors, chemokines, and cytokines with paracrine and autocrine activities), immunomodulatory (T-cells, dendritic cells, and natural killer cells), and anti-inflammatory effects [23]. These factors suppress the local immune system, inhibit fibrosis and apoptosis, enhance angiogenesis, and stimulate proliferation and differentiation. Iwai et al. [24] demonstrated that local injection of adipose tissue-derived MSCs facilitated attenuation of fibrosis.

The normal wound healing process includes endothelial injury, myofibroblast activation, macrophage migration, inflammatory signal stimulation, immune activation, matrix deposition, and remodeling. Especially in the first 24–28 h, many molecular reactions occur in the tissue. Fibroblasts are crucial elements in the inflammation process. Moreover, a functional microcirculatory bed is of critical importance for the prevention of epithelial loss and fibrosis [25]. Fibrosis is one of the most common and refractory pathological processes. Fibrosis is a redundant accumulation of extracellular matrix (ECM) in tissues by collagen reaction, and at the end of the recovery process, a thick fibrotic neocapsule can develop. MSCs can directly release hepatocyte growth factor and bone morphogenetic protein-7, which are important inhibitors of fibrosis. MSCs have been shown to exert antifibrotic effects in animal models by matrix metalloproteinases [26]. Unlike synthetic meshes, autologous MSCs are immune compatible, which is an advantage in the remodeling process.

In the present study, the granulation and inflammation scores in the kidney specimens were similar between the control and omentum repair groups. However, they were significantly lower in the omentum repair group than in the primary repair groups. This finding suggests that the omentum attenuates granulation and inflammation related to kidney injury. Transposition of autologous omentum may act by reducing macrophage infiltration and fibrosis.

In many studies, histological damage to the kidneys has been evaluated in tissues with the endothelial, glomerular, tubular, and interstitial (EGTI) scoring system [27]. This scoring system considers histological damage in 4 individual components (endothelial, glomerular, tubular, and interstitial) and is scored from 0 to 4. This scoring is performed in the renal cortex, especially for glomerular units. Therefore, we preferred to use a new scoring system for our histopathological evaluation so that it was possible to evaluate different components of regeneration in all of the kidney tissue.

There was a trend toward a decrease in urea and creatinine levels in the study groups. Additionally, there was no correlation between urea and creatinine levels and histological scores. These findings can be explained by the fact that we could not produce sufficient nephron damage with our trauma model. In the future, this experiment will be repeated with major kidney tissue damage. Contrary to our results, Garcia-Gomez et al*.* [12] reported that omentum was effective for the treatment of kidney injuries. In the context of the use of the omentum, progression to chronic kidney disease could be reduced in a rat model [12]. However, in that study, the kidney injuries were larger (5/6 subtotal nephrectomy).

According to the results of the present study, granulation and inflammation in kidney specimens were positively correlated with granulation, inflammation, fibrosis, and foreign body reaction in the surrounding tissue. Healing process completion in the kidney specimen was inversely correlated with granulation and foreign body reaction in the surrounding tissue. As expected, inflammation in the surrounding tissue was positively correlated with granulation, fibrosis, and foreign body reaction in the surrounding tissue. Moreover, fibrosis in the surrounding tissue was positively correlated with inflammation and foreign body reaction. Therefore, inflammation and granulation may lead to fibrosis, and interventions to reduce inflammation and granulation after injury may aid in the prevention of fibrosis and permanent tissue damage.

Granulation in the kidney specimen was strongly and positively correlated with inflammation and foreign body reaction in the kidney specimen and strongly and negatively correlated with the healing process completion score in the kidney specimen. Moreover, inflammation in the kidney specimen was positively correlated with granulation and foreign body reaction in the kidney specimen and negatively correlated with the healing process completion score in the kidney specimen. Therefore, we can speculate that inflammation and granulation after injury are also related to a reduced healing capacity, and efforts to reduce inflammation may also aid in the acceleration of healing.

The limitations of this study were that only blood creatinine and urea levels were used for biochemical evaluation of the renal injury and we did not measure the urine concentrations due to the technical inadequacy of urine collection from rats. Moreover, this study only included a qualitative histopathological analysis and lacked a kidney injury group without primary repair or omentum repair. The use of such a group might improve the quality of the evaluation of the effect of the primary repair and omentum repair. Last, the injury model used in this study did not cause an increase in urea or creatinine levels. Therefore, performing a similar study with a larger kidney injury model would provide a better evaluation of these interventions.

## Conclusion

We aimed to determine the repair capacity of omental tissue in renal injury in a rat model. According to our results, the use of autologous omentum tissue for repair of kidney injury attenuated inflammation and granulation compared with primary repair. These results imply that the use of omental tissue to facilitate healing of kidney injury may theoretically lead to a more effective healing process and reduced fibrosis and tissue and functional loss.

## Data Availability

We agree that the materials described in the manuscript, including all relevant raw data, will be freely available to any scientist wishing to use them for noncommercial purposes without breaching participant confidentiality.

## Abbreviations

AAST: American Association for the Surgery of Trauma
ECM: Extracellular matrix
H&E: Hematoxylin–eosin
MSCs: Mesenchymal stem cells
MTC: Masson’s trichrome
NO: Nitric oxide
SD: Standard deviation
VEGF: Vascular endothelial growth factor
